# Optimised Desorption Electrospray Ionisation Mass Spectrometry Imaging (DESI-MSI) for the Analysis of Proteins/Peptides Directly from Tissue Sections on a Travelling Wave Ion Mobility Q-ToF

**DOI:** 10.1007/s13361-018-2049-0

**Published:** 2018-08-30

**Authors:** Mark W. Towers, Tamas Karancsi, Emrys A. Jones, Steven D. Pringle, Emmanuelle Claude

**Affiliations:** 1 Waters Corporation, Stamford Avenue, Altricham Road, Wilmslow, SK9 4AX UK; 2 Waters Research Centre, Záhony utca., C ép., 1. Em., Budapest, 1031 Hungary

**Keywords:** DESI, Desorption electrospray ionisation, MSI, Mass spectrometry imaging, Peptides, Proteins, IMS, Ion mobility separation

## Abstract

Desorption electrospray ionisation mass spectrometry imaging (DESI-MSI) is typically known for the ionisation of small molecules such as lipids and metabolites, in singly charged form. Here we present a method that allows the direct detection of proteins and peptides in multiply charged forms directly from tissue sections by DESI. Utilising a heated mass spectrometer inlet capillary, combined with ion mobility separation (IMS), the conditions with regard to solvent composition, nebulising gas flow, and solvent flow rate have been explored and optimised. Without the use of ion mobility separation prior to mass spectrometry analysis, only the most abundant charge series were observed. In addition to the dominant haemoglobin subunit(s) related trend line in the *m*/*z* vs drift time (DT) 2D plot, trend lines were found relating to background solvent peaks, residual lipids and, more importantly, small proteins/large peptides of lower abundance. These small proteins/peptides were observed with charge states from 1+ to 12+, the majority of which could only be resolved from the background when using IMS. By extracting charge series from the 2D *m*/*z* vs DT plot, a number of proteins could be tentatively assigned by accurate mass. Tissue images were acquired with a pixel size of 150 μm showing a marked improvement in protein image resolution compared to other liquid-based ambient imaging techniques such as liquid extraction surface analysis (LESA) and continuous-flow liquid microjunction surface sampling probe (LMJ-SSP) imaging.

**Electronic supplementary material:**

The online version of this article (10.1007/s13361-018-2049-0) contains supplementary material, which is available to authorized users.

**Graphical Abstract:**
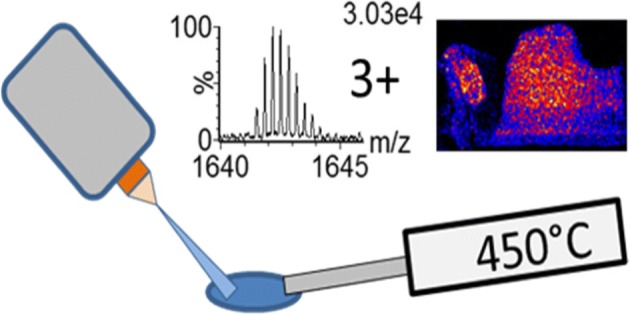
ᅟ

## Introduction

Mass spectrometry imaging [1] (MSI) allows for the spatial mapping of a wide range of molecules from a sample surface. Since the introduction of desorption electrospray ionisation (DESI) in 2004 [2], this technique has gained popularity for use in MSI applications. This is primarily due to the lack of sample preparation compared to matrix-assisted laser desorption ionisation (MALDI) and its applicability to the analysis of a wide variety of molecules [3–5] including lipids. However, whilst DESI has been shown to be useful for the analysis of proteins [6–11] and tryptic peptides [12] deposited on sample surfaces, it has been widely regarded as being unable to obtain protein/peptide signals by MSI from biological tissues [13, 14]. Takats et al. [15] postulate that the ionisation mechanism for peptides and proteins in DESI must be one that involves the formation of charged droplets, as the formation of multiply charged ions is thought to exclude gas phase-ionisation mechanisms. Additionally, they reported optimised parameters for the ionisation of peptides from spotted samples; these included a short sprayer tip to surface distance with high gas and liquid flow rates. It was suggested that the impact of charged droplets on to the surface is essential for the ionisation of peptides [15]. The optimised parameters were shown to be significantly different compared to those obtained for explosives, lipids and aromatic hydrocarbons. Furthermore, the spectra generated for peptides were similar to what would be expected via electrospray analysis, i.e. multiply charged ions were generated by DESI. The ionisation method described to be most applicable for proteins is the “droplet pickup” method [15–19]. In this process, an initial spray generates a pool of liquid on the surface to be analysed, into which the analyte molecules are extracted. Subsequent charged droplets from the electrospray sprayer collide with the pool resulting in the ejection of secondary charged droplets containing the analyte, leading to an electrospray like ionisation process, with ion sampling via the inlet capillary. The desorption characteristics of the analytes seem to be highly dependent on the substrate. This may explain the inherent difficulty observing proteins from biological tissue matrices by DESI imaging. All other DESI based protein work performed to date being from glass, PTFE or PMMA spotted surfaces [6–11].

Currently, MALDI is the preferred ionisation method of analysis for intact proteins by MSI [1]. However, MALDI predominantly generates singly charged ions and so for large proteins and peptides, this requires an instrument with a high mass to charge (*m*/*z*) range such as an axial time of flight instrument. Furthermore, it can mean that analysis of proteins using MALDI is poorly suited to quadrupole based instruments such as hybrid quadrupole time of flight (Q-TOF) mass spectrometers that may have transmission limitations due to the *m*/*z* transmission window of the quadrupole. In the majority of cases, MALDI is performed under vacuum and requires extensive sample pre-treatment such as the application of matrix to the sample surface. Recently, ambient techniques such as liquid extraction surface analysis [20] (LESA) and continuous-flow liquid microjunction surface sampling probe analysis [21] (LMJ-SSP) (recently commercialised as the Flowprobe by Prosolia, USA) have been shown to be applicable to the MSI of proteins from tissues [14, 22]. Similarly to DESI, these techniques use an electrospray ionisation process and produce multiply charged ions allowing the detection of higher molecular weight analytes within a smaller *m*/*z* range. However, both LESA and LMJ-SSP (typically) have image resolution limitations due to the size of the contact point. This is especially true in the case of LESA with the sampling being conducted from discrete locations. Recent advances in raster mode continuous flow LMJ-SSP by Griffiths et al. [22] utilising the commercially available Flowprobe source have improved the achievable lateral pixel size, reporting images achieved with a 50-μm pixel width; the pixel height was still limited to 600 μm (it should be noted that there is however a high degree of lateral convolution due to the 600 μm contact size) [22]. Another variant of LMJ-SSP developed by Laskin and co-workers [23, 24] called nano-DESI has recently claimed to have a lateral resolution of 200 μm. This was achieved using a home built nano-DESI imaging source by Hsu et al. via a continuous acquisition mode [13].

DESI-MSI suffers from some lateral convolution, being a continuous raster technique; the sampling area is significantly smaller than with LMJ-SSP and LESA and in the same region as nano-DESI setup of Hsu et al.

It has previously been shown that ion mobility separation (IMS) can be highly beneficial when analysing complex samples in the context of an MSI experiment. This is particularly true when dealing with overlapping signals such as multiply charged series. Škrášková et al. [25] demonstrated the use of travelling wave IMS combined with DESI analysis to image and identify gangliosides in murine brain by extracting *m*/*z* IMS drift time (DT) multiply charged trend lines to separate them from the more abundant singly charged lipid and background ions. Additionally, Feider et al. [5] presented the use of high-field asymmetric waveform ion mobility separation (FAIMS) for a semi-selective enhancement of the charge series of cardiolipins and gangliosides using DESI as well as a number of proteins by LMJ-SSP. The use of FAIMS mass spectrometry coupled to LESA for enhanced spatial profiling of proteins in mouse brain and liver was described in a study by Griffiths et al. [14].

Here we show the optimisation and performance of DESI for the imaging of intact proteins directly from tissue sections, utilising a custom built heated sampling capillary with a commercial DESI source. Also demonstrated is the use of travelling wave IMS to enable detection and isolation of various protein charge series which would otherwise remain buried in the noise and thus be undetectable without this additional mode of separation.

## Methods

### Tissue Sectioning

Rat liver sections used were ethically sourced and surplus to requirement from a previous study. Briefly, rat liver organ was frozen at − 80 °C and sectioned at a thickness of approximately 12 μm using a cryostat (Leica Microsystems, Wetzlar, Germany) and thaw mounted on to a glass slide followed by storage at − 80 °C.

### Tissue Washing

To aid with protein ionisation efficiency, the lipids and salts were removed and proteins fixed by washing the tissue prior to analysis [26–28]. The tissue sections were immersed for 1 min in the following solutions: 70% ethanol aq, 90% ethanol aq, and 100% ethanol. Liver sections were then immersed in chloroform for 25 s. The tissue sections were then dried in a vacuum desiccator for approximately 10 min or until residual chloroform had been removed.

### Source Setup—Sprayer and Heated MS-Inlet Capillary

Experiments were performed on a Waters SYNAPT G2-*Si* ion mobility enabled Q-ToF mass spectrometer (Waters Corporation, UK). A modified 2D-DESI source (Prosolia, USA) was used to acquire the data. The DESI sprayer was modified to isolate the main body from the capillary voltage. This was achieved by passing a pre-cut 2.5 in (360 μm outer diameter, 20 μm inner diameter) taper tip emitter (Waters, Milford, USA) through the length of the sprayer body, then using a T-junction microcoupling to apply the voltage to the solvent and connect the fused silica solvent line to the emitter. Applying the voltage to the emitter only and not the sprayer body improved stability when using an increased level of water in the solvent composition.

In order to aid in the desolvation of secondary droplets, the standard MS-inlet capillary was replaced with a heated MS capillary. Briefly, the modified heated capillary consisted of a stainless steel inlet tube coved by a fibreglass jacket and a nickel heating wire wound around the jacket covered in a ceramic paste. Finally, a second fibre glass jacket was added to enclose the heating coil. The nickel heating coil was connected to a Switch mode power supply (72-830A, Tenma). A photograph of the sprayer setup and the custom inlet capillary is seen in Online resource 1.

The temperature of the capillary could be adjusted between ambient temperature and approximately 500 °C by adjusting the voltage and therefore current applied to the heater (between 0 and 14 V). A calibration curve was generated for the heated capillary by measuring the temperature with a thermocouple for a given voltage and current. This could be used to approximate the temperature applied under experimental conditions.

### DESI Source Geometry Setup

Initial setup of the DESI source was performed by the analysis of lipids from unwashed tissue using a solvent flow rate of 2 μL/min; a solvent composition of 98% methanol, 0.1% formic acid, 1.9% water; a gas pressure of 6 Bar; a spray voltage of 4.5 kV; and a spray angle of 65°.

The emitter capillary protrusion was initially optimised by observing the removal of material from a patch of black ink on a glass slide. The area etched in the ink was indicative of both the size and direction of the spray. The capillary protrusion was optimised to ensure a tightly focused spray spot. The sprayer body was rotated to ensure the correct orientation and hence directionality of the spray solvent into the surface of the sample. The spectra were then observed to ensure the presence of lipids from the tissue. The solvent composition was subsequently changed to 80% acetonitrile, 19.8% water and 0.2% formic acid. Further optimisation of the geometry was then performed on washed liver tissue to maximise the appearance of multiply charged peaks from the haemoglobin subunits (alpha-1/2 and beta-2).

### Experimental Conditions

Multiple parameters were explored to improve the desorption and ionisation of proteins directly from tissue sections such as nebulising gas pressure (2 to 7 Bar), solvent flow rate (1 to 4 μL/min), spray voltage (2.5 to 5 kV), inlet capillary temperature (25 to 430 °C) and finally spray solvent compositions; comprising either acetonitrile or methanol, with water and 0.2% formic acid (50 to 90% organic content). To assess the effects of the varying analytical conditions, 4.0 mm wide × 1.0 mm height lines were acquired from washed rat liver tissue with a scan time of 1.5 s, a DESI stage velocity of 100 μm/s and a longitudinal line spacing of 150 μm. The resulting spectra were summed to give an overall measure of the signal. Data were acquired using tandem ion mobility separation and oa-ToF mass spectrometry.

### Instrument Settings

The instrument was operated in sensitivity mode at a resolution of >10,000 (FWHM). Mass spectra were acquired across the range of *m*/*z* 100 to 2000 for optimisation experiments and an extended range to *m*/*z* 2500 for final full tissue imaging experiments. The key settings for the ion mobility/trap and transfer cell were as follows: Trap DC bias 45, IMS wave velocity linear ramp mode, start velocity 1100 m/s, end velocity 300 m/s and transfer wave velocity 137 m/s.

### Data Acquisition and Processing

**Figure 1:**
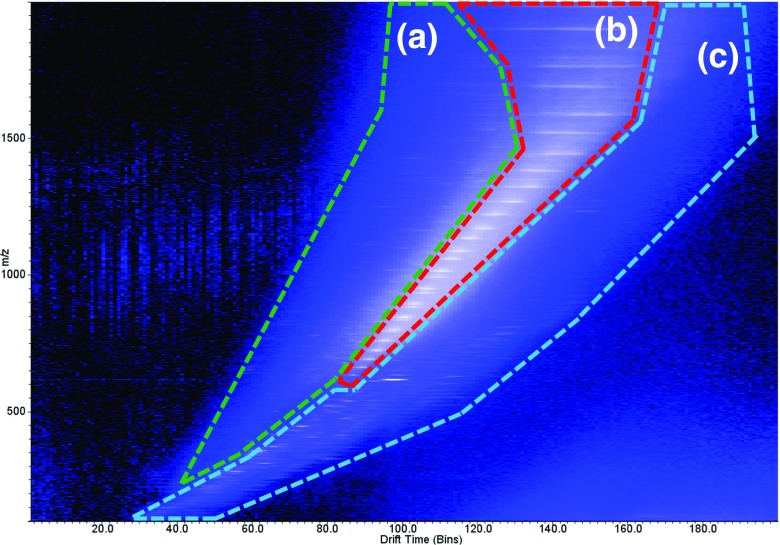
Typical IMS plot from an optimised setup on liver tissue. Three distinct regions can be observed: (**a**) high velocity region, multiply charged peptides and small proteins; (**b**) haemoglobin trend line; (**c**) solvent peaks, remaining lipids and other small molecules (singly charged)

Acquisition setup, processing and visualisation of imaging data were performed using High Definition Imaging (HDI) 1.4 (Waters Corporation, UK). Data were acquired using MassLynx 4.1 (Waters Corporation, UK). Ion mobility data trend line extraction was performed using DriftScope 2.9 (Waters Corporation, UK) with visualisation of spectra in Masslynx 4.1. To judge the effect of changing parameters, the signal for a number of selected peaks was monitored. Two regions were extracted using DriftScope; a high velocity trend line (Figure 1, region A) and the haemoglobin subunits (alpha-1/2 and beta-2) trend line (Figure 1, region B). The summed spectra were baseline subtracted. The relative intensity of each peak across each condition and the mean relative intensity were calculated to give a global average for those peaks for each condition. The selected peaks for region A were *m*/*z* 948, 1016, 1161, 1354 and 1499 with charge states from 4+ to 8+. The selected peaks for the haemoglobin subunits (region B) were the haemoglobin subunit alpha-1 peaks at *m*/*z* 854, 1086, 1267, 1520 and 1900 covering charge states from 8+ to 18+. For the generation of images via HDI, individual charge state trend lines were extracted from the whole tissue data set using Driftscope. The average *m*/*z* and drift time for each charge state was calculated in MassLynx. The imaging data set was then processed via HDI using a target list, with an extraction window of 0.2 Da and a drift window of two bins. The generated charge state images were then summed to give an overall distribution for the protein.

## Results and Discussion

### The Utilisation of the Heated Capillary and Ion Mobility for the Detection of Proteins

The analysis of intact proteins directly from tissue sections has proven to be problematical using DESI. Whilst optimisation can easily be achieved from protein standards spotted on Teflon targets, and several previous publications detail this approach [6, 8, 15], these optimised settings do not translate well to direct analysis from tissue sections. This stems from issues regarding the desorption of protein related ions directly from the tissue or differences in desorption time scales compared to glass or Teflon [15–19].

Through the use of a custom built heated capillary, it proved possible to detect highly abundant multiply charged ion series. Charge states from 9+ to 22+ were observed. Due to the obvious visual presentation of the tissue and expected abundance, these peaks were presumed to belong to haemoglobin subunits.

The haemoglobin subunit charge series was used as a target to optimise the other parameters. After optimisation of all the parameters, the haemoglobin subunits could be observed without the use of the heating element of the heated capillary (approximately 25 °C at inlet orifice). However, the haemoglobin subunit peaks were observed with a lower intensity. This was most prevalent for the lower charge states where the average intensity increase for the 8+ to 12+ charge states of haemoglobin subunit alpha-1 was ×8.5 (± 1.3 SD) with heating. For the higher charge states (13+ to 20+) the average increase was ×2.9 (± 1.0 SD). Furthermore, more significant differences were observed in the lower abundant proteins and those with a high IMS velocity/ lower collisional cross section. Due to their lower intensity and high background chemical noise level, IMS was required to extract these species. It is noted that without the addition of the heated capillary, these species were not observed even using ion mobility separation. Figure 1 shows an example of an IMS drift time vs *m*/*z* 2D plot that was acquired with the heated capillary after optimisation. A number of trend lines can be seen in the 2D plot relating to different molecular species. In the high velocity/highest ion mobility region (region A), a number of multiply charged intact peptides and small proteins can be observed. The central nested region of the plot (region B) is occupied by the charge series of a mixture of haemoglobin subunits; the lowest ion mobility region (region C) contains a mixture of singly charged solvent peaks, lipids and other small molecules. From the 2D plot, spectra for regions A to C were extracted to create new raw files maintaining the drift time dimension whilst summing the retention time dimension.

**Figure 2:**
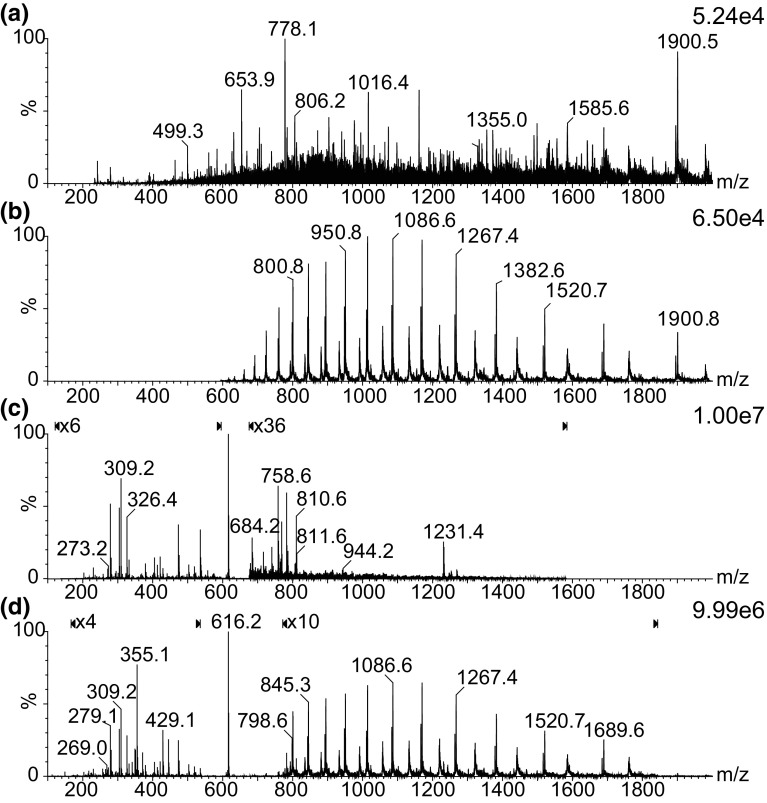
(**a**–**c**) Summed spectra extracted from drift plot regions (a–c) in Fig. 1. (**d**) Complete data set

Figure 2 shows IMS extracted spectra for each region (Figure 2A–C), including a mass spectrum obtained from the complete data set where the IMS dimension is summed (Figure 2D). Due to the high intensity of the haem peak (*m*/*z* 616) from haemoglobin, additional magnifications have been applied to several parts of the MS spectra in Figure 2C, D. Whilst the haemoglobin subunits charge state envelope, from region B, can be seen without the use of ion mobility, the majority of the low intensity peaks from region A are obscured.

**Figure 3:**
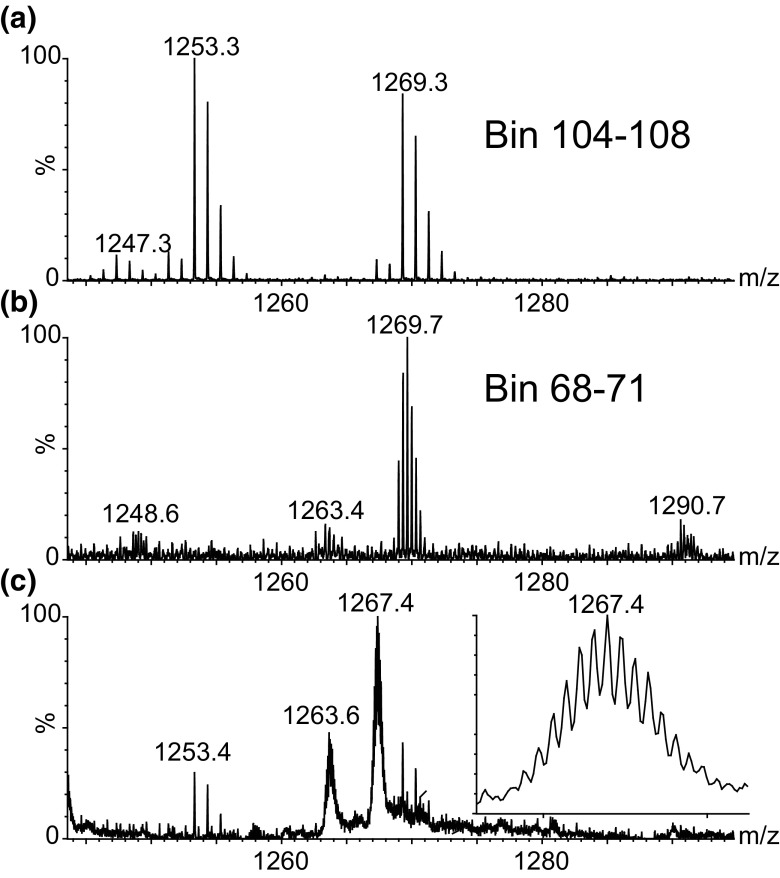
Example showing the benefit of ion mobility for resolving spectral complexity/overlapping signals: (**a**) combination of IMS bins 104–108; (**b**) combination of bins 68–71; (**c**) combination of the full 200 bins (no IMS separation); inset—expansion of the 12+ haemoglobin subunit alpha-1 peak

This is further highlighted in Figure 3; the *m*/*z* region from *m*/*z* 1240 to 1300 was extracted, and the lower spectra (Figure 3C) show the data without using ion mobility separation (combined spectrum of all IMS bins). Looking through the mobility dimension, additional peaks which are obscured in the non-mobility separated spectrum can be observed. The top spectrum (Figure 3A) shows the summation of bins 104–108 with two singly charged peaks which are only partially visible in the bottom spectrum. The middle spectrum (Figure 3B) is the combination of bins 68–71 and shows a triply charged peak which has the same *m*/*z* as the *m*/*z* 1269 peak in the top spectrum and is not visible in the bottom spectrum being completely lost under the other peaks.

**Figure 4:**
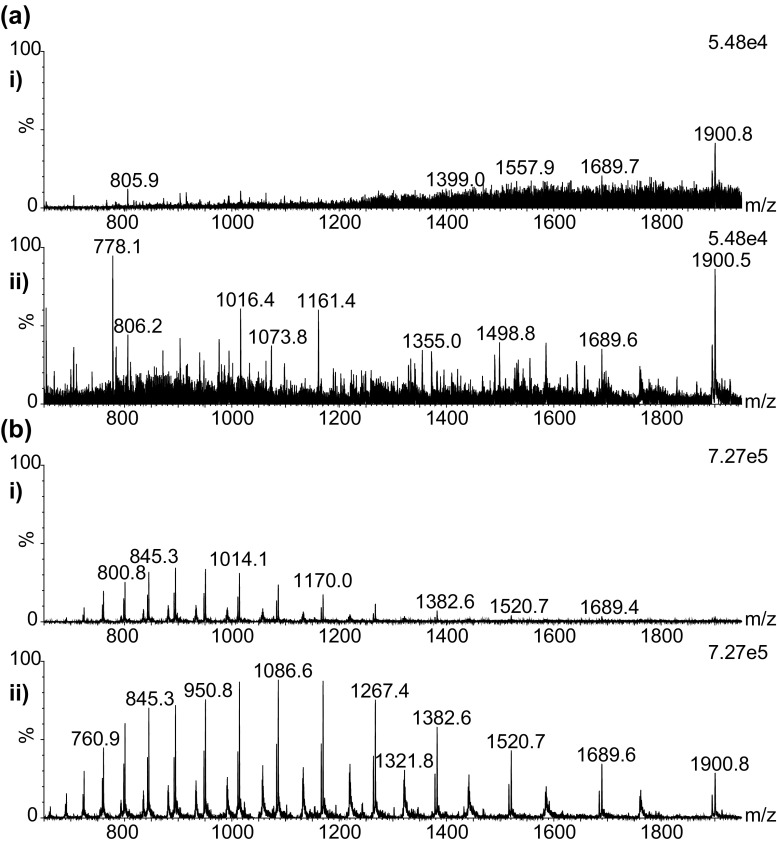
Spectra extracted from regions a and b (**a** and **b**, respectively) without heating i (0v on inlet ≈ 25 °C) and with heating ii (12v on inlet ≈ 430 °C). Baseline subtracted with linked vertical axis

Figure 4A, B shows the effect of heating the MS inlet capillary on the MS signals detected in region A and B. The top spectrum (i) in each case shows data acquired without the heated capillary and the bottom spectrum (ii) with the capillary at approximately 430 °C. It can be seen that whilst under optimised condition the heated capillary is not essential for the detection of highly abundant protein species such as the haemoglobin subunits, it is required for the lower abundance multiply-charged species observed within the high velocity trend line A.

### Solvent Composition

**Figure 5:**
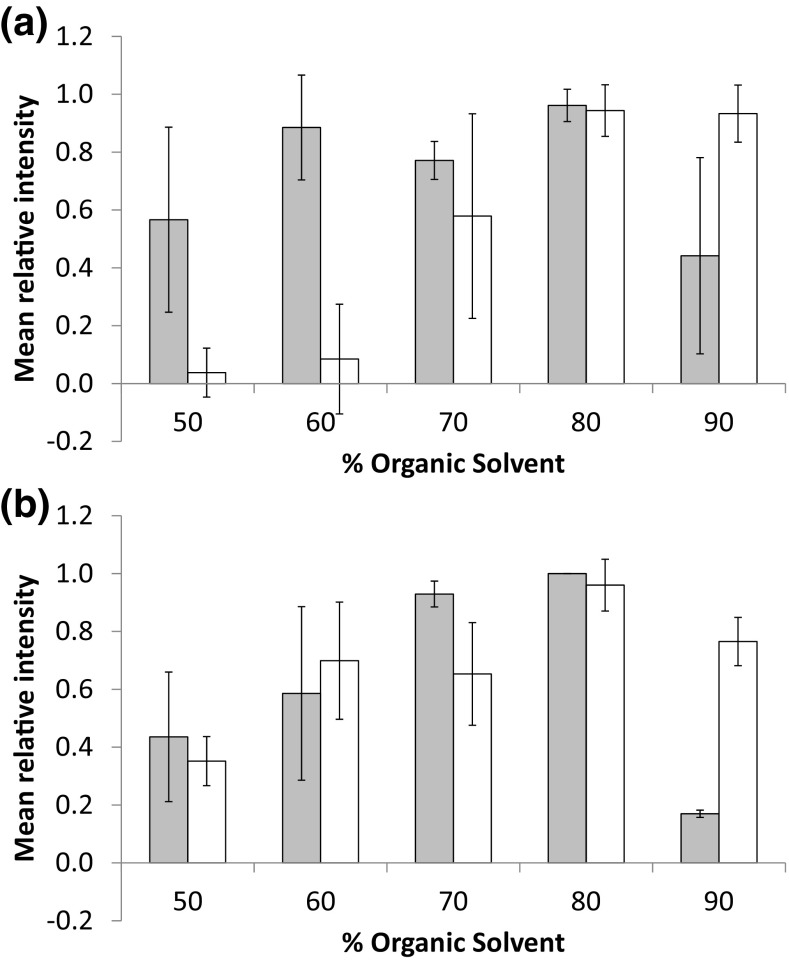
Effect of adjusting the organic composition of spray solvent (acetonitrile—grey bars, methanol—white bars) on selected peaks within the high velocity region (**a**) and haemoglobin region (**b**)

Typically for the analysis of lipids by DESI, a mixture of methanol and water (with optional formic acid for positive ion mode analysis) is used as the DESI spray solvent. With the current sprayer configuration, the optimum methanol-water mixtures lie in the range between 90 and 98% methanol [29]. Usually for protein/peptide work in LC-MS/direct infusion experiments, mixtures of acetonitrile and water are used, typically in the region of 30 to 50% acetonitrile. Here we have assessed different mixtures of methanol and acetonitrile in an effort to optimise desorption of the proteins/peptides from the tissue sections. For both the acetonitrile and methanol compositions, a range of 50 to 90% organic solvent compositions were tested. To assess the effect of the solvent composition, the intensity of selected peaks within the high velocity region A and haemoglobin subunit region B, trend lines were monitored. The summed spectra were baseline subtracted; the relative intensity of each peak across each condition and the mean relative intensity were calculated to give a global average for those peaks for each condition. The results for the high velocity region A peaks are displayed in Figure 5A whilst the haemoglobin subunit peaks (region B) are displayed in Figure 5B.

In both cases, 80% of organic solvent in the spray was optimum for the detection of peaks of the haemoglobin subunits from the liver sample. A drop in signal was observed with 90% organic solvent, which was particularly severe for the acetonitrile mixture. Whilst reasonable intensities were observed for the haemoglobin subunits peaks at 60 and 70%, a sharp decrease was observed in the number of peaks within the higher velocity peptide/protein region A particularly with the increased methanol composition.

The two optimum solvent systems were compared using the same tissue on the same day, and it was found that the 80% acetonitrile composition outperformed the 80% methanol composition with regard to the peak intensity of the higher velocity peaks.

### Nebulising Gas and Solvent Flow Rate

**Figure 6:**
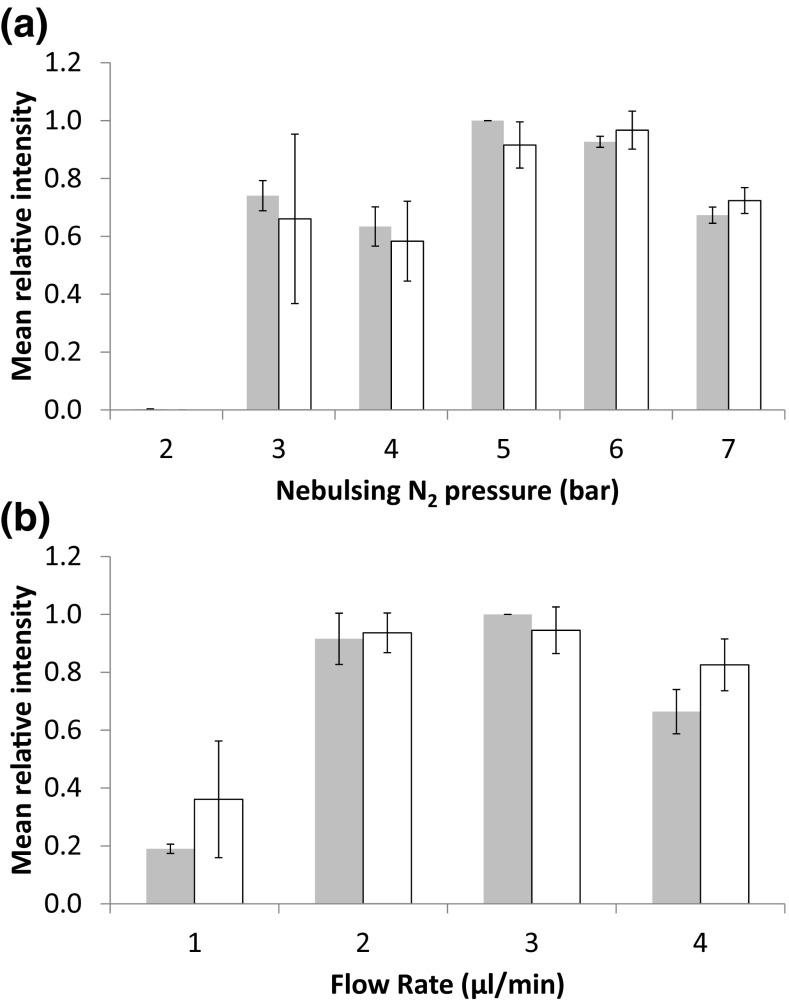
Effect of modulating nebuliser gas pressure (**a**) and solvent flow rate (**b**) on selected peaks for the high velocity region (grey) and haemoglobin region (white)

In addition to the solvent composition, the effect of the nebulising gas pressure and flow rate was assessed. The gas pressure was modulated between 2 and 7 Bar with a fixed flow rate of 3 μL/min. The flow rate was varied between 1 and 4 μL/min using a 5-Bar gas pressure. In both cases, a solvent composition of 80:19.8:0.2 acetonitrile:water:formic acid was used. The same peaks were monitored as with the solvent composition optimisation. Optimal gas pressure was found to be between 5 and 6 Bar (Figure 6A), with an optimal flow rate of between 2 and 3 μL/min (Figure 6B).

### MSI of Complete Liver Tissue Section

**Figure 7:**
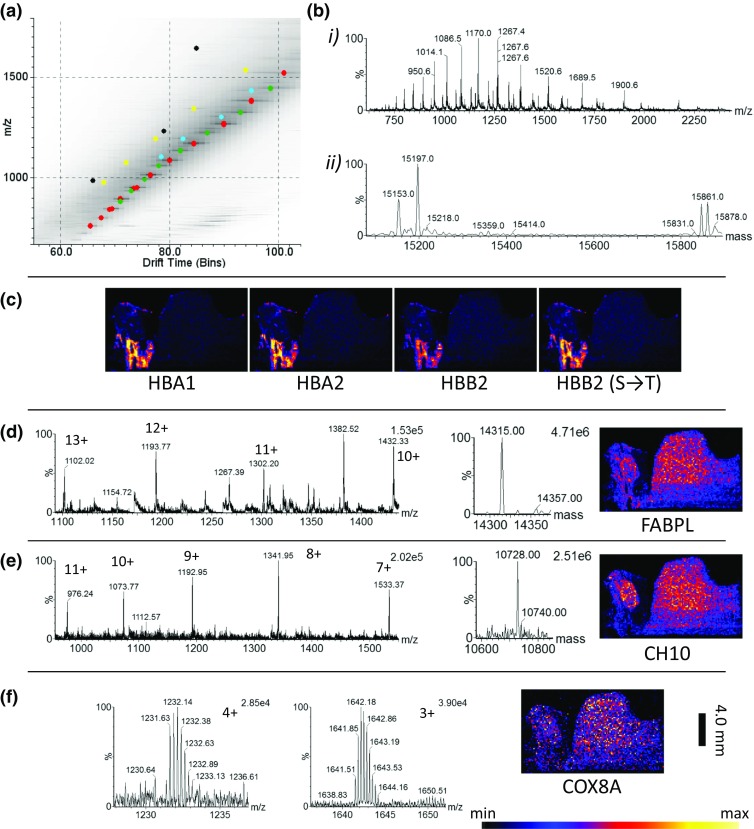
Results of protein imaging of a complete liver tissue section. (**a**) 2D drift vs *m*/*z* plot (square root scale) with trend lines highlighted (HBA 1 and 2); red, haemoglobin subunit alpha-1/2 related; green, haemoglobin subunit beta-2 related (HBB2); blue, fatty acid binding protein (FABPL); yellow, 10 kDa heat shock protein (CH10); black, cytochrome c oxidase subunit 8A (COX8A). (**b**) (i) Extracted haemoglobin trend line; (ii) result of MaxEnt 1 algorithm deconvoluted spectrum i, showing haemoglobin related peaks. (**c**) Composite distributions for haemoglobin charge series. (**d**) Extracted trend line, MaxEnt 1 spectrum and combined ion image related to FABPL. (**e**) Extracted trend line, MaxEnt 1 spectrum and combined ion image related to CH10. (**f**) 4+ and 3+ charge states related and combined ion distribution image for COX8A (scale bar 4 mm)

DESI imaging analysis of complete tissue sections was performed at a pixel size of 150 μm using the optimised parameters. The haemoglobin subunit trend line and high velocity trend line were extracted maintaining the ion mobility separation; by examining these trend lines, a number of charge state series could be observed. The charge state series were isolated from the drift plot to allow the maximum entropy algorithm [30, 31] (MaxEnt 1 – MassLynx, Waters) to be used and the average mass of the protein calculated. The calculated drift times and average masses for the different charge states were calculated and used to generate images to be displayed in HDI 1.4. Figure 7A shows an expanded view of the drift plot with the extracted charge state series highlighted.

Figure 7B (i) shows the dominant haemoglobin subunit-related series, after performing MaxEnt 1 deconvolution MS spectrum analysis (Figure 7B (ii)); a number of peaks can be observed, based on accurate mass; these have been assigned as haemoglobin subunit alpha-1 and haemoglobin subunit alpha-2 (aspartic acid to alanine substitution) [32, 33] UniProtKB—P01946 (HBA_RAT) [34] (red highlighted trend line) and haemoglobin subunit beta-1 with one of its natural variants with a serine substituted for a threonine—UniprotKB—P02091 (HBB1_RAT) [34] (green highlighted trend line). The highlighted peaks were extracted, and the images were combined to generate the distributions seen in Figure 7C. Close to the haemoglobin subunits trend line, an additional charge series was observed (highlighted blue); the extracted trend line, MaxEnt 1 spectrum and combined image are seen in Figure 7D*.* This series has tentatively been assigned as a fatty acid binding protein (liver) with an acetylation [35, 36], UniProtKB—P02692 (FABPL_RAT) [34]. A trend line relating to a 10-kDa protein (highlighted yellow) is seen in Figure 7E which could potentially be a 10-kDa heat shock protein with an unknown modification—P26772 (CH10_RAT). Based on literature, this is normally observed with a N-acetylation [37, 38]. This would however result in a mass deficit of −84 Da between the observed and the calculated, so would need to be investigated further by either a bottom up or top down methods. Figure 7F shows two species relating to a small protein with a deconvoluted average mass matching cytochrome c oxidase subunit 8A—P80433 (COX8A_RAT), after transit peptide cleavage [39] (highligted black). Whilst the tissue appears relatively homogenous, some different distributions can be observed. A clear margin can be observed around the tissue indicating that delocalisation has not occurred at 150 μm pixel size.

**Figure 8:**
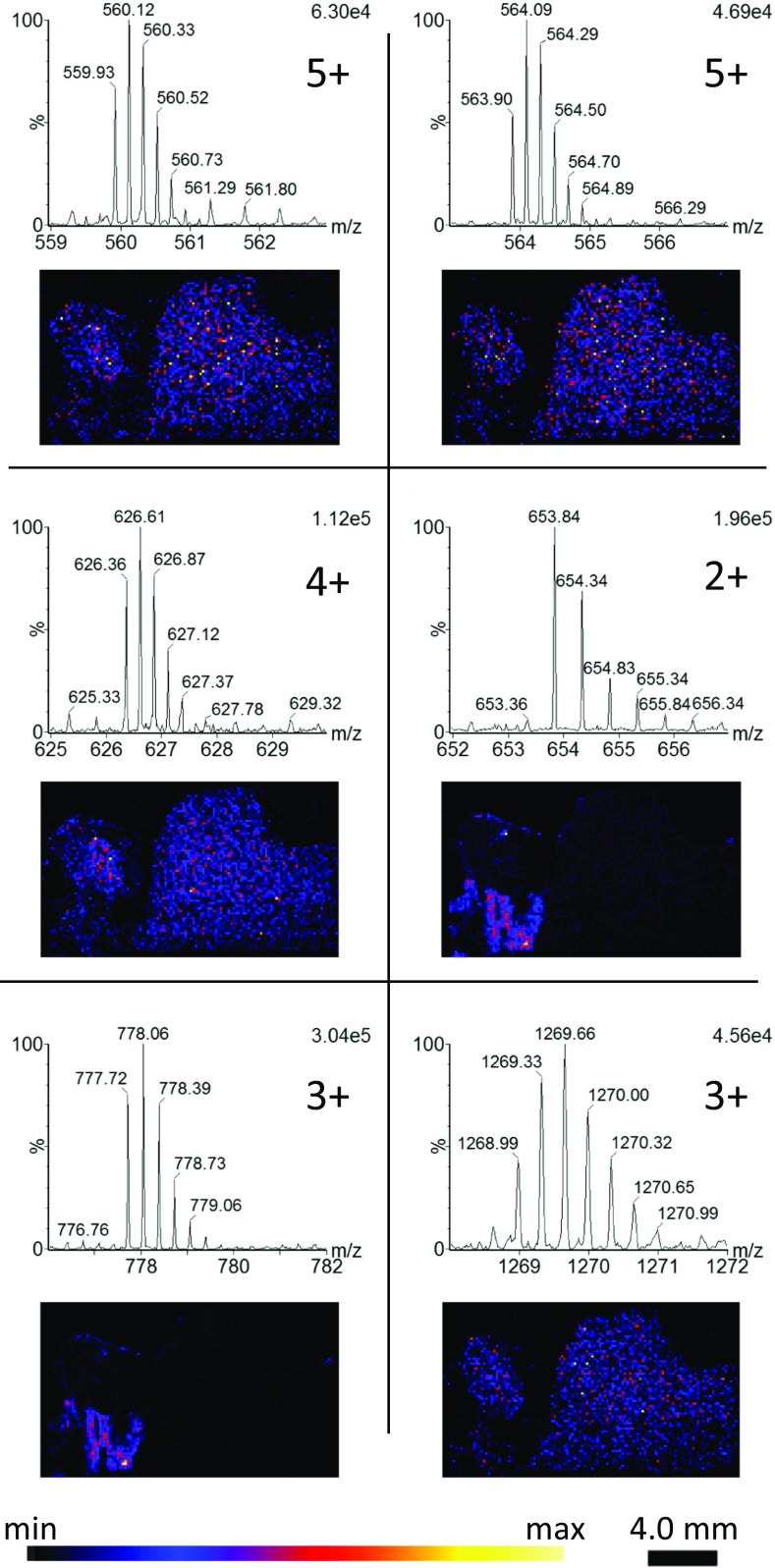
Mobility extracted endogenous peptide peaks with corresponding distributions (sum of isotopes)

In addition to the protein charge series, a number of endogenous peptides were also observed with charge states ranging from 2+ to 5+ (Figure 8).

The peptides can be seen with differing distributions, with some present within the regions showing high levels of the haemoglobin subunits suggesting they may be serum-based peptides as opposed to the tissue located peptides. The observation of peptides also suggests that the same conditions for proteins should be equally applicable to digested tissue sections.

## Conclusions

This work has shown for the first time the utilisation of DESI for the detection of proteins and peptides directly from a tissue section combined with imaging mass spectrometry. The combination of DESI with ion mobility separation enabled significant improvements in the signal to noise and allowed the separation of highly complex overlapping signals. The implementation and optimisation of the heated inlet capillary have been described, allowing the optimisation of solvent composition, solvent flow rate and nebulisation gas pressure for the modified DESI interface. With this optimised interface, it has been possible to observe the distribution of multiple intact peptides/protein signals within the tissue at substantially improved pixel resolution (150 μm) compared to other liquid extraction techniques. Obviously, there is an inherent difficulty in assigning identifications to peaks by accurate mass alone due to the wide variety of documented/undocumented post-translational modifications, whilst continued development of the DESI technology and interface should bring further improvements in both the sensitivity and the pixel resolution; further work will need to be performed in order to investigate top-down and bottom-up strategies for the identification of proteins directly from tissue by DESI. This development does however show an interesting new avenue of investigation complimentary to current DESI and other MSI techniques.

## Electronic Supplementary Material

ESM 1 (PDF 119 kb)
