# Supplementary material for: Optimised Desorption Electrospray Ionisation Mass Spectrometry Imaging (DESI-MSI) for the Analysis of Proteins/Peptides Directly from Tissue Sections on a Travelling Wave Ion Mobility Q-ToF
Source: J Am Soc Mass Spectrom. 2018 Aug 30;29(12):2456–66. doi: 10.1007/s13361-018-2049-0 (PMC6276080; doi:10.1007/s13361-018-2049-0)
Supplement: Supplementary file 1 — (PDF 119 kb) [file 13361_2018_2049_MOESM1_ESM.pdf]

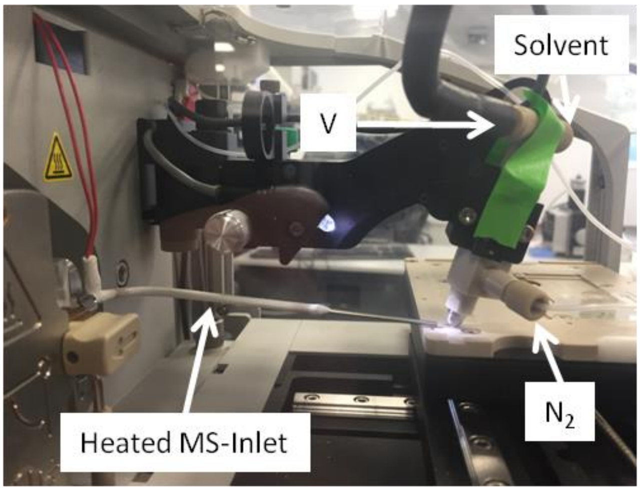

On line resource 1. Modified sprayer setup, showing additional t-junction isolating the voltage from the sprayer body and the heated MS-inlet.
